# Early Neuropathic Treatment May Prevent the Chronic Stage of Complex Regional Pain Syndrome Type II (CRPS II​)

**DOI:** 10.7759/cureus.36861

**Published:** 2023-03-29

**Authors:** Arianna J Caradonna, Danielle Lee, Moorice Caparó

**Affiliations:** 1 Physical Medicine and Rehabilitation, Albert Einstein College of Medicine, Bronx, USA; 2 Neurology, Montefiore Medical Center, Bronx, USA; 3 Physical Medicine and Rehabilitation, Montefiore Medical Center, Bronx, USA

**Keywords:** neuropathic pain treatment, neuropathic pain syndrome, crps type 2, crps-2, crps ii, complex regional pain syndrome, crps

## Abstract

Complex regional pain syndrome (CRPS) is a debilitating condition characterized by autonomic and inflammatory features, often caused by fractures, surgeries, or other injuries. Multimodal treatment is utilized, which often includes neuropathic medications and physical therapy. We had a case of a 24-year-old man who was diagnosed with CRPS II following an open reduction and internal fixation of a trimalleolar fracture. Significant improvement of edema, pain, and function was achieved with early initiation of gabapentin, nortriptyline, and physical therapy. In this case report, we discuss the therapeutic challenges regarding his recovery and review the literature on the utility of medications and interventional methods in treating CRPS II. We note that early treatment response may be an important prognostic indicator for the progression of CRPS II and additional studies targeting interventions for the specific type and clinical stage of CRPS are needed.

## Introduction

Complex regional pain syndrome (CRPS) is defined as a chronic pain condition in which the pain experienced is significantly disproportionate in degree or time to the typical course of any known trauma or other lesions. The pain often starts in patients’ distal extremities and often has abnormal motor, sensory, sudomotor, vasomotor edema, and/or trophic findings [1]. The prevalence of CRPS is low and ranges from 5.4-26.2 per 100,000 persons per year [2]. Its pathogenesis is poorly understood, and many patients suffer a poor quality of life due to a lack of successful therapeutic strategies [3].

CRPS is divided into two types: CRPS I (formerly, reflex sympathetic dystrophy) and CRPS II (formerly, causalgia). In CRPS I, there is no apparent nerve injury whereas in CRPS II, there is an identifiable nerve injury [4,5]. In the clinical course of CRPS, there is an acute stage followed by a chronic stage. In the acute phase, patients typically experience trophic changes, edema, erythema, a burning sensation, increased temperature, and decreased range of motion. In the chronic (cold) phase of CRPS, patients experience clammy and cyanosed limbs due to excessive vasoconstriction [6].

We present the case of a male patient who developed CRPS II following an open reduction internal fixation (ORIF) after an ankle fracture and was treated shortly after his surgery with several modalities, including neuropathic medications and physical therapy, resulting in a successful resolution of his symptoms by five months post-op.

This article was previously presented as a meeting abstract at the 38th AAPM Annual Meeting on March 18, 2022.

## Case presentation

**Figure 1:**
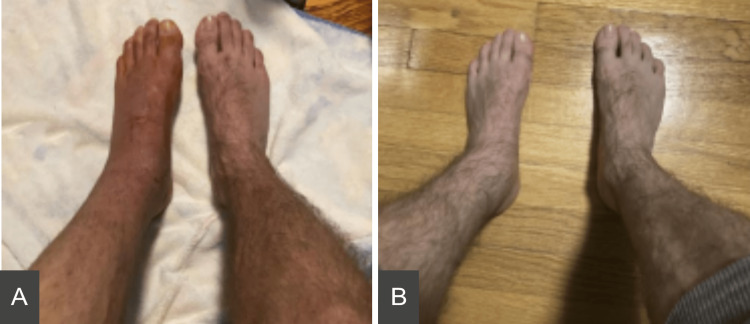
Pictures showing the patient’s physical exam one month post-op (A) and four months post-op (B).

A 24-year-old man with no past medical history presented to the Emergency Department after sustaining a traumatic fall causing a tibiotalar dislocation, distal fibular fracture, posterior malleolar fracture, and a ruptured deltoid ligament. He underwent an urgent trimalleolar ORIF with a posterior approach. At one month post-op, he developed new onset plantar numbness, allodynia, and dysesthesia that felt like a sensation of “stepping on a lego” which exacerbated at night, for which he was started on gabapentin 100mg TID and referred to pain management. Examination showed a well-healing wound without signs of infection but was notable for swelling and erythema of the left ankle; dry, scaling skin in the distal tibial joint; decreased sensation to light touch on the medial, dorsal, and plantar surfaces of the left foot; hyperesthesia of the metatarsal heads; and decreased hair growth on the dorsal aspect of the left foot (Figure 1A), not better explained by other diagnoses. The provisional diagnosis of CRPS was made using the Budapest criteria [7]. The patient was increased to gabapentin 300 mg three times a day. He also started physical therapy, consisting of contrast baths, desensitization techniques, and strengthening exercises. At two months post-op, an electromyography (EMG) showed that both the left sural sensory nerve and left lateral plantar motor nerve had no response. Left medial plantar motor and left superficial peroneal sensory nerves showed reduced amplitude. The EMG results favored the diagnosis of CRPS II. An X-ray of the left foot with three views showed a healing fracture and no dislocation. MRI lumbar spine was unremarkable. A triple-phase bone scan of the calves, ankles, and feet showed an abnormal radiotracer uptake in all three phases in the left ankle. The patient’s pain persisted, interfering with his sleep, and gabapentin was uptitrated to 600 mg three times a day. Nortriptyline 50 mg nightly was also initiated. The patient continued physical therapy three times per week.

At three months post-op, his pain improved with gabapentin and nortriptyline, but he continued to have sporadic flare-ups which required Percocet and Motrin as needed. At four months post-op, the physical exam showed marked improvement in erythema, swelling, or decreased hair growth in his left ankle and foot (Figure 1B). The patient expressed interest in tapering down the medications, and his gabapentin was decreased to 600 mg twice daily, and he continued taking nortriptyline 50 mg daily. At five months post-op, he achieved near-complete resolution of pain and returned to jogging on a treadmill. By this time, the patient was successfully titrated off both medications. Unfortunately, the patient was lost to follow-up at the pain clinic after this visit. At eight months post-op, the patient’s therapy slowed down and he unfortunately developed contractures of his left flexor hallucis longus tendon, for which the removal of hardware and posterior capsulotomy was indicated.

## Discussion

CRPS remains a complex multifactorial disease and therefore data for the treatment of this disease remains very limited despite recent advancements in the research of CRPS. One challenge is the issue of diagnostic heterogeneity, which can have important implications for both prognosis and treatment. This is further complicated by the dynamic nature of CRPS and how its presentation can evolve over time [8]. CRPS I and II currently do not differ in treatment choice and there is a lack of evidence on whether treatment responses to intervention differ based on subtype of the disease.

Several mechanisms have been proposed in the development of CRPS. Neuropathic inflammation may play an integral role in its development, as evidenced by the upregulation of Substance P and calcitonin gene-related peptide (CGRP) in affected tissues and the upregulation of A-δ nociceptive nerve fibers in patients with CRPS [9]. A coupling between the sympathetic and peripheral nociceptive nervous systems may develop over time.

While early recognition and a multimodal approach are the mainstays of treatment, no gold standard treatment modality is known to alter the natural course of CRPS. Multiple drug treatments have been tried, but unfortunately most trials have not been tested in double-blinded, randomized, controlled trials (RCT), making their evidence insufficient [10]. Some drug treatments include but are not limited to anti-inflammatories, immune-modulators, anticonvulsant or neuropathic medications, and opioids. Several studies support that gabapentin and amitriptyline are relatively safe and effective in improving pain to various degrees and reducing the sensory deficit in the affected limb in CRPS I [11,12].

Tricyclic antidepressants are thought to be helpful especially with their added benefits of antidepressant properties. Evidence is limited for the use of opioids for CRPS. One RCT showed no difference in pain reduction in patients using morphine compared to placebo after eight days of use [13]. The use of opioids for an extended period is also known to worsen allodynia and therefore this must be carefully considered when treating a disease that is characterized by hyperalgesia [10].

More recently, interventional therapies such as sympathetic nerve blocks and dorsal root ganglion stimulation have been trialed in CRPS and proven to be effective [14]. These therapies may be considered in patients who are refractory to conservative treatments. Further studies are needed to determine the criteria for which subset of patients are deemed appropriate candidates for interventional treatment.

One limitation of this case report is that EMG findings were not repeated once the patient’s symptoms improved. Thus, we cannot confirm if the patient’s nerve injuries improved. Further studies are needed to delineate the difference in the pathophysiology of CRPS I and II and to determine when invasive treatments over conventional drug treatments should be considered to provide the most effective treatment. This case highlights that early initiation of a neuropathic agent with suspected CRPS II may play a key role in preventing the chronological changes seen in CRPS II.

## Conclusions

Evidence for the treatment of CRPS is limited due to few RCT studies. This case highlights that early intervention targeting neuropathic pain may play a significant role in the chronification and progression of CRPS II. In a time when pain medicine is becoming interventional, it is important to still consider oral pharmacological therapy as a method for early intervention. Further studies are needed to understand the pathophysiologic changes that occur in CRPS II. A high-power study comparing the efficacy of early and late initiation of medications may be helpful.
